# A study of the risk of mental retardation among children of pregnant women who have attempted suicide by means of a drug overdose

**DOI:** 10.5249/jivr.v4i1.85

**Published:** 2012-01

**Authors:** Dora Petik, Barbara Czeizel, Ferenc Bánhidy, Andrew E. Czeizel

**Affiliations:** ^a^Foundation for the Community Control of Hereditary Diseases, Budapest, Hungary.; ^*b*^Second Department of Obstetrics and Gynecology, Semmelweis University, School of Medicine, Budapest, Hungary.

## Abstract

**Background:**

The aim of the study was to estimate the effect on the fetal development of high doses of prescription drugs taken as a suicide attempt during pregnancy.

**Methods:**

Pregnant women were identified among self-poisoned females in the toxicological inpatient clinic in Budapest between 1960 and 1993. Congenital abnormalities, intrauterine development based on birth weight and post-conceptional age, mental retardation, cognitive-behavioral status were compared in exposed children born to mothers who had attempted suicide by means of a drug overdose during pregnancy with their siblings, born either before or after the affected pregnancy, as sib controls.

**Results:**

Of a total of 1 044 pregnant women, 74 used the combination of amobarbital, glutethimide and promethazine (Tardyl®, one of the most popular drugs for treatment of insomnia in Hungary) for suicide attempt. Of these 74 women, 27 delivered live-born babies. The mean dose of Tardyl® used for suicide attempts was 24 times the usually prescribed clinical dose. The rate of congenital abnormalities and intrauterine retardation was not higher in exposed children than in their sib controls. However, of the 27 exposed children, eight (29.6%) were mentally retarded (Χ${\mathrm{_{{1}}^{{2}}}}$)=79.7, p= Sig) while mental retardation did not occur among 46 sib controls. These exposed children were born to mothers who attempted suicide with Tardyl® between the 14th and 20th post-conceptional weeks. The components of Tardyl® used separately for a suicide attempt during pregnancy were not associated with a higher risk of mental retardation. Therefore the high doses of Tardyl® associated with the high risk for mental retardation may be due to the interaction of its three drug components.

**Conclusions:**

The findings of the study showed that the high doses of a drug containing three components may be associated with a significantly increased risk for mental retardation without any structural defects, whereas each of these three component drugs taken alone was not associated with this adverse effect.

## Introduction

Suicide and self-inflicted injury are classified as intentional causes of death or diseases.^1-4^Hungary led the world in suicide mortality with the rates of about 45 per 100 000 persons in the 1970s and 1980s, later there was a decrease in this rate but it has remained high in an international perspective. In addition, the rate of suicide attempts by means of prescription drugs has increased significantly worldwide.^3,4^Suicide attempts by means of drugs and other chemicals have been termed self-poisoning.^6^The recent self-poisoning epidemic has produced a major socio-medical problem, mostly among young females.^7^Such suicide attempts also occur among pregnant women.^8,9^It is of interest that the number of pregnant survivors of suicides has increased significantly as a result of more effective medical intervention. However, survival may be associated with a greater risk of congenital abnormalities and/or mental retardation in the fetuses/children.

We have evaluated the potential to estimate the teratogenic/fetotoxic risk of these prescription drugs^10^Clinical trials conducted before approval and marketing of a drug generally do not include pregnant women. Thus, it is necessary to base the potential for human teratogenic/fetotoxic risk on the results of experimental animal investigations. Ideally, screening tests in laboratory animals would identify the doses of drugs that can be human reproductive or developmental toxicants/teratogens. However, current screening systems are imperfect, and multiple factors prevent direct extrapolation of results on pregnant women. Thus, the harsh reality is that humans are the ultimate test model for detection of drugs and specially the doses of drugs that are found to be human teratogens.

Two types of post-marketing data of drugs have been used to estimate their human teratogenic potential. The first type of data set is obtained through case reports, clinical case series, and randomized controlled trials. However, case reports have serious selection bias, clinical case series usually do not have appropriate controls, and there are serious ethical barriers to performing randomized controlled trials on pregnant women. The second type of data set is associated with analytical epidemiological studies and/or registry/surveillance/monitoring systems.^11^However, identifying a possible association between the low clinical doses of drugs and structural birth defects, i.e. congenital abnormalities are confounded by the recall bias of mothers with affected children compared to mothers with healthy babies, and there is usually an inability to estimate a dose-response relationship. Although such post-marketing data can be useful in predicting human teratogenic risk of drug exposures, medical practitioners must consider the accuracy of these predictions with caution.

Thus the self-poisoning model of pregnant women based on the Budapest Registry of Self-poisoned Patients^12^offers a unique approach for studying the potential teratogenic and fetotoxic-neurotoxic effects of drugs on the fetuses. Of 1 044 self-poisoned pregnant women, 411 delivered live-born infants between 1960 and 1993, and of these 411 children, 367 (89.3%) were evaluated for health status, in particular congenital abnormalities, birth weight and post-conceptional age, cognitive status as well as behavioral development.^10^

This paper summarizes the data of 27 children who were born to mothers who used the combination of amobarbital, glutethimide and promethazine (Tardyl®) for a suicide attempt during pregnancy. This medicinal product is one of the most popular prescription drugs for the treatment of insomnia in Hungary. Of these 27 children, eight (29.6%) were mentally retarded.

## Methods

Budapest and the surrounding area have a population of about three million people. All self-poisoned patients in this area were admitted to the Department of Toxicological Internal Medicine, Korányi Hospital, Budapest.^12^The objective of the study was to identify pregnant women among self-poisoned females and to evaluate the effect of large doses of drugs on their exposed children. Gestational age was calculated from the first day of the last menstrual period, however, we used the term post-conceptional pregnancy age, estimated from the first day of the third week of the first lunar (28 day) pregnancy month, i.e., from the speculative day of conception. Thus the usual duration of pregnancy was 266 days and 38 pregnancy weeks.

The study included three steps. First, self-poisoned pregnant women were identified among female patients in the Department of Toxicological Internal Medicine, Korányi Hospital, and their pregnancies were confirmed by gynecological examination. Each self-poisoned pregnant woman had a personal card including personal, medical, lifestyle data as well as all information regarding the self-poisoning in the study pregnancy. Doses and acute effects of the drugs used for self-poisoning were based on data obtained from three sources: (i) information obtained from the pregnant women; (ii) drug levels present in their blood and (iii) the clinical picture of intoxication. Clinical intoxication was defined as mild (not comatose at or after admission), moderate (comatose or unconscious at or after admission), severe (unconsciousness longer than one day after admission and/or need for artificial respiration), very severe (life-threatening, i.e., unconsciousness more than two days with severe complications such as uremia or multi-organ failure) and fatal. However, pregnant women whose suicides had had a fatal outcome were excluded from the study because their fetuses could not be evaluated.

The study protocol was evaluated first by the Institutional Ethical Review Board, but because this methodological approach was unusual, it was forwarded to the Central Ethical Committee of Ministry of Health. This committee finally approved the study protocol with 3 criteria: (i) Self-poisoned pregnant women had to sign a consent form regarding their voluntary participation in the study and granting permission for follow-up home visits and examination of their children. (ii) Self-poisoning pregnant women had a right to refuse the collaboration at any time during the study. (iii) We had to organize a special high standard prenatal care and delivery service for self-poisoned pregnant women who decided to continue their pregnancies.

Secondly, all surviving self-poisoned pregnant women were visited at home after the expected day of their deliveries to elucidate their pregnancy outcomes. Data regarding their miscarriages and still- and live-births (including birth weight and gestational age) were medically recorded in their discharge summaries because all deliveries and clinically recognized miscarriages took place in inpatient obstetric clinics and the birth attendants were obstetricians at the time of the study period. In addition, the mothers with their live-born children were invited for a thorough medical and psychometric examination at our institute.

Thirdly, all exposed children and their sibs/siblings were examined by a pediatrician, medical geneticist and psychologist according to the protocol of the study. The aim of detailed medical examination was the detection of congenital abnormalities (i.e. structural birth defects) and minor anomalies (unusual morphologic variants without serious medical consequences to the children). Chromosome evaluation (karyotyping) was undertaken for children with multiple defects and/or mental retardation. The diagnosis of fetal alcohol syndrome (FAS) was based on a semi-quantitative score.^13^Autopsy records were available for the evaluation of deceased children. The cognitive development of children was measured by the help of the Hungarian Developmental Test^14^used routinely in Hungary. Children were classified into 4 groups according to their intelligence quotient (IQ): 1) above mean (110-120 IQ), 2) mean (90-109 IQ), 3) under mean (70-89 IQ) and 4) mental retardation (less than 70 IQ). There were two diagnostic criteria of mental retardation: (i) less than 70 IQ , and (ii) children were not able to attend normal primary school.^15^All exposed children with suspected mental retardation were followed until school age, when they were examined by official experts and they were referred to special schools for retarded students. The behavioral status of children was estimated by the psychologist using the Behavioral Style Questionnaire.^16^

Mothers, who could not visit our institute, were visited at home by the pediatrician and psychologist to examine exposed children and their sibs according the study protocol. Visits of families to our institute and the visits to the home of exposed children were used to check and to complete personal and lifestyle data of mothers that were collected on hospital records. Socioeconomic status of mothers was estimated based on employment status and educational level, and they were classified into three classes: high, medium and low. Smoking was classified according to the number of cigarettes smoked daily. Drinking habits were estimated on the information provided by the mothers and classified as abstinent, occasional (less than one drink per week), regular (from one drink per week to one drink/day) and hard (more than one drink/day) drinkers. If the quantity of drinking changed during the study pregnancy, we recorded the maximum.

The major methodological challenge was to find appropriate controls for exposed children, and finally we used familial and population controls. The familial controls comprised of the previous and subsequent unexposed child(ren) of self-poisoned pregnant women and they were called sib controls for comparison with the exposed child. (If pregnant women repeated suicide attempts during the study period, their live-born babies were evaluated as exposed children, but these exposed children were not evaluated as sibs.) The medical condition, including birth defects and mental retardation, in addition to the cognitive status and behavioral scale of the sib controls were examined and diagnosed at the same time and place (our institute or their home) as of exposed children by the same protocol and by the same experts.

Population controls included 38 151 newborns without birth defects in the Hungarian Case-Control Surveillance of Congenital Abnormalities, 1980-1996,^17^they were used as a reference sample (representing 1.8% of all Hungarian births) for the comparison of exposed children.

Details of these materials and methods, in addition to the characteristics of self-poisoned pregnant women were described previously.^10,9^

The pregnant women evaluated in this paper attempted suicide with Tardyl® alone or in combination with other drugs. Tardyl® (EGIS) contains 125 mg amobarbital, 125 mg glutethimide, and 7.5 mg promethazine in one tablet, and most of these pregnant women used it as a hypnotic drug.

At the statistical analysis of data, the SAS version 8.02 statistical software package was used (SAS Institute, Cary, NC). Quantitative variables of pregnant women and their children were evaluated by the Student t test and categorical variables by the chi square test. The prevalence at birth of congenital abnormalities and mental retardation in exposed children was compared with their unexposed sib controls, and odds ratios (OR) with 95% confidence interval (CI) were calculated by an unconditional multiple logistic regression model.

## Results

Of 1044 self-poisoned pregnant women in the total sample, 74 (7.1%) used Tardyl® for their suicide attempt. Of these 74 pregnant women, one had a false address and one refused to participate, 27 decided to terminate their pregnancy (based upon socioeconomic reasons affecting their unplanned and unwanted pregnancies; in addition to the suspected teratogenic effects of the drugs used for their suicide attempts), 18 pregnancies ended in fetal death (very early loss: 12; miscarriage: 6) and 27 pregnant women delivered live-born babies. Thus, of 367 live-born and evaluated exposed children, 27 (7.4%) belonged to the Tardyl® sample.

The characteristics of the 27 pregnant women who attempted suicide with Tardyl® and delivered live-born babies were compared to the Hungarian reference pregnant sample (Table 1). The mean age is lower among mothers who attempted suicide because of a larger proportion of the youngest (19 years or less) age group (37.0% vs. 8.6%), though their mean birth order was not significantly lower. The lower proportion of married women and higher proportion of low socioeconomic status were also characteristic for pregnant women who attempted suicide. There was a 2.8- and 18.5-fold higher rate of smokers and regular/hard drinkers, respectively, among self-poisoned pregnant women. Congenital abnormalities and mentally retardation did not occur among the self-poisoned pregnant women (the latter conclusion is based on their schooling and personal communication with us), i.e. the mothers of exposed children.

**Table:** Table 1: **Main variables of 27 pregnant women who attempted suicide during pregnancy with Tardyl® (amobarbital 125 mg, glutethimide 125 mg, promethazine 7.5 mg in one tablet) alone or with other drugs and delivered live-born babies, and a reference sample of Hungarian pregnant women who delivered newborns without birth defects in the Hungarian Case-Control Surveillance of Congenital Abnormalities, 1980-1996^17^**

Variables	Tardyl®(N=27)		Hungarian pregnant women(N=38,151)		Comparison	
Age, yr (mean, S.D.)	22.4	5.4	25.4	4.9	t=2.9;	P=0.004
Birth order (mean, S.D.)	1.6	0.9	1.7	0.9	t=0.9;	p=0.35
Married (%)	63.0		96.1		x ${\mathrm{_{{1}}^{{2}}}}$=79.7	P<0.0001
Socioeconomic status (%)						
High	7.4		38.0			
Medium	22.2		30.6		x ${\mathrm{_{{2}}^{{2}}}}$=20.3	P<0.0001
Low	70.4		31.4			
Smoker (%)	51.9		18.9*			
Regular/hard drinker (%)	29.6		1.6*			

*These figures were based on a sub-sample in which 3,022 mothers were visited at home and data were obtained by cross interview of family members^29^

The number of Tardyl® tablets taken by the 27 self-poisoned pregnant women ranged from 10 to 60 tablets with a mean of 24.1 + 11.5 tablets. Of these 27 pregnant women, eight combined Tardyl® with other drugs, mainly benzodiazepines, for their suicide attempt. The drug intoxication was classified as very severe in three, severe in 19, moderate in two and mild in three pregnant women.

**Figure 1:Family tree of two exposed children born to a mother who attempted suicide with Tardyl® in their two pregnancies.**
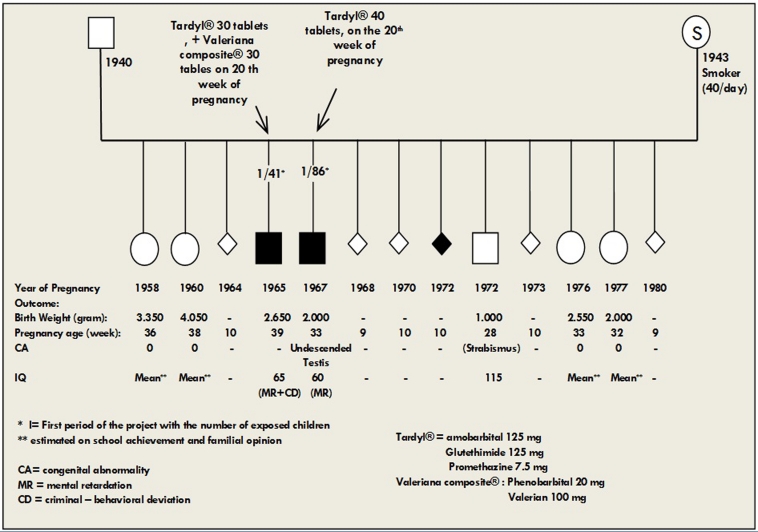


Of 27 exposed newborns, 14 (51.9%) were male. The data of birth outcomes of the 27 exposed children and their 46 unexposed sibs are shown in Table 2. These data were available for all exposed children and unexposed sibs. Two exposed children (7.4%) had congenital abnormalities, namely undescended left testicle and a multiple defect diagnosed as FAS by us. The mother of the boy affected with the undescended testis attempted suicide using 40 tablets of Tardyl® in the 20th pregnancy week (Figure 1), however, the critical period of undescended testis is during the last months of pregnancy. The exposed boy with FAS had a mild microcephaly (his first year head circumference was 40 cm) and three minor anomalies (flat occiput, smooth philtrum, thin upper lip) and his IQ was 65 and 70 in two different measurements. His hard drinker mother had a panic disorder, and she attempted suicide with 20 tablets of Tardyl® and 10 tablets of glutethimide (2,500 mg) on the 16th post-conceptional week.

**Table:** Table 2: **Comparison of birth outcomes in exposed children born to pregnant women who attempted suicide with Tardyl® during pregnancy and in their unexposed sibs**

Variables	Exposed children(N=27)		Unexposed sibs(N=46)		Comparison	
Categorical	No.	%	No.	%	OR	95% CI
Congenital abnormalities	2	7.4	2	4.4	1.8	0.2 – 13.9
Mental retardation	8	29.6	0	0.0	Χ${\mathrm{_{{1}}^{{2}}}}$=79.3	p<0.0001
Low birth weight (less than 2500 g)	4	14.8	7	15.2	1.0	0.3 – 3.8
Preterm birth (less than 35th week)	6	22.2	8	17.4	1.6	0.6 – 5.5
Quantitative	Mean	S.D.	Mean	S.D.	t =	p =
Birth weight (g)	2,883	55	2,895	67	0.10	0.94
Pregnancy age (wk)	36.4	2.8	36.8	2.9	0.08	0.79

Bold numbers show significant association

Six pregnant women used high doses of Tardyl® for their suicide attempt between the 3rd and 8th post-conceptional weeks, i.e., the critical period for most major congenital abnormalities; however, these six exposed children did not have any defect.

Of 46 sibs, two (4.4%) were affected with defects: oesophageal atresia with tracheal fistula and FAS (he was the sib of the previously mentioned exposed child with FAS). Thus, the prevalence at birth of congenital abnormalities was not higher in exposed children than in their unexposed sibs (Table 2).

Mean birth weight was similar in exposed children and their sibs (Table 2)although both had lower values than the mean birth weight of the reference Hungarian newborns (3,276 + 511 gram). Pregnancy age at delivery also did not show a significant difference between exposed children and their sibs, which was also shorter than the Hungarian population figure (37.4 + 2.0 week). Thus, the pregnancy week specific birth weights did not indicate any intrauterine fetal growth retardation. The rate of low birth weight and preterm birth was also similar in exposed children and in their sibs.

Of 27 exposed children, eight (29.6%) had the diagnosis of mental retardation and they attended a special school for mentally retarded children. In general their IQs were measured twice or more with nearly similar findings. Only two exposed children: a boy with FAS (who was mentioned previously) and a girl had once 65 IQ and on another measurement 70 IQ. The main data of these eight exposed children are shown in Table 3. Of their eight mothers, six used only Tardyl® for their suicide attempt, three took 20 tablets. (One box of Tardyl® contains 20 tablets) Of these eight pregnant women, one was classified as hard and five as regular drinkers, but we were able to diagnose FAS only in one exposed child (and his sib) of a hard-drinker mother. Of these eight exposed children, six had normal karyotypes; the other two children were without any visible defects but were housed in a special institution and thus chromosome examination was not allowed. Other syndromes were also not identified in these exposed children.

**Table:** Table 3: **Data of exposed children with mental retardation and moderate behavioral deviation**

Period/Number	Suicide attempt				Exposed children			IQ	Mental retardation		
	Tardyl (tbl)	Other drugs	Pregnancy Age (wk)	Sex	Birth weight(g)	Pregnancy age (wk)	Defect (Minor anomaly)		Karyo-type	Age of diag-nosis(yr)	Estimated etiology
I/41	30	Yes*	20	M	2,650	39	0 (0)	65	46,XY	15	Tardyl phenobarbital
I/86	40	0	20	M	2,000	33	Undescended testicles (0)	60	46,XY	13	Tardyl®
II/29	20	AA	18	F	2,850	35	0 (NE)	65	NE	6	Tardyl®
II/81	30	AA	18	M	2,550	36	0 (NE)	55	NE	6	Tardyl®
II/107	40	0	14	F	2,750	37	0 (NE)	65,70	NE	6	Tardyl®
III/107	20	AA	17	F	2,550	37	0 (enamel hypoplasia)	55	46,XX	6	Tardyl®
III/572	20	Yes*	16	M	2,500	38	FAS	65,70	46,XY	6	Tardyl®+Glutethimide/alcohol
III/603	20	AA	16	F	1,500	30	0 (low broad nasal bridge, umbilical hernia)	60	46,XX	6	Tardyl®
									Moderate behavioral deviation		
II/91	20	0	21	F	2,400	31	0 (NE)	85	NE	6	Tardyl®
III/185	20	AA	28	F	3,450	39	0 (low broad nasal bridge, enamel hypoplasia)	85	46,XX	6	Tardyl®

* Valeriana compsitaR (Phenobarbital 600 mg + Valeriana 3.000 mg). AA= alcohol abuse and suicide attempt together M= Male, F= Female / FAS= Fetal alcohol syndrome / NE= no examination

**Table:** Continue table 3: **Data of exposed children with mental retardation and moderate behavioral deviation**

Behavioral deviation	Mother				Remarks ***
	Age(yr)	Drinking	Smoking (cig/d)	Health	
Severe	21	0	40	Repeated suicide	6 sibs: one of them I/86**,another sib: 115 IQ
NE	24	0	40	Repeated suicide	6 sibs: one of them I/41**,another sib: 115 IQ
Severe	19	Regular	20	-	EC in foster home,1 sib
Severe	19	Regular	20	-	EC in foster home,no sib
Mild	28	0	0	-	2 sibs,one: 100 IQ
Severe	18	Regular	30	Repeated suicide	1 sib
Moderate	25	Hard	20	Panic disorder	2 sibs: 100 IQ,85 IQ
Severe	22	Regular	10	-	2 sibs
Moderate	24	0	20	-	2 sibs: 100 IQ;85 IQ
Moderate	22	Regular	0	-	2 sibs,one: 100 IQ

** Glutethimide 2.500 mg *** See Figure 1

Eight mentally retarded exposed children had 20 sibs, but two were excluded as exposed children because their mothers attempted suicide in two different pregnancies (Figure 1). The mother of these two exposed children was not a drinker, but was a heavy smoker and her children showed an association between the maternal suicide attempt with Tardyl® during pregnancy and some neurotoxic effects of the high doses of Tardyl®. Of her five unexposed children, four had normal cognitive status, and the IQ was measured as 115 in the fifth child, i.e. her son. However, the two exposed children were mentally retarded, and one of them had been placed in a young offenders institution for repeated offences by the time he was 15 years old.

Of the unexposed 46 sibs, none had mental retardation (Table 2); all sibs attended normal primary schools.

The cognitive status and behavioral scale measurements were planned for all exposed children and their unexposed sibs, but IQ could only be tested in 22 exposed children and 20 unexposed sibs, while the behavioral scale was measured in 16 exposed children and 16 unexposed sibs (Table 4). The calculated mean IQ was lower in exposed children than in their unexposed sibs. The timing of suicide attempts showed that the high doses of Tardyl® were associated with a high risk for mental retardation when exposure occurred between the 14th and 20th post-conceptional weeks, i.e. in the second trimester of pregnancy (Table 5).

**Table:** Table 4: **Comparison of cognitive status and behavioral scale in exposed children born to pregnant women who attempted suicide with Tardyl® during pregnancy and in their unexposed sibs **

Cognitive status (IQ)	Exposed children(N=22)	Unexposed sibs(N=20)	Behavioral scale	Exposed children(N=16)	Unexposed sibs(N=16)
Above mean (110-120)	3	4	Normal	4	9
Mean (90-109)	7	12	Mild	4	6
Under mean (70-89)	4	4	Moderate	3	1
Mental retardation (less than 70)	8	0	Severe	5	0
Mean ± S.D.	82.2 ± 20.0(t=3.8)	100.0 ± 9.7(p=0.04)	Distribution	χ${\mathrm{_{{3}}^{{2}}}}$ = 8.3	p=0.03

**Table:** Table 5: **Association between the occurrence of different categories of cognitive status in exposed children and the time of suicide attempts with Tardyl® according to pregnancy age periods, in addition distribution of different categories of cognitive status in exposed children and their sibs according to the drinking habit of self-poisoned pregnant women **

Cognitive status (Estimated mean IQ)	Pregnancy age periods (wk)			Self-poisoned pregnant women			
	7 12 (N=8)	13 24 (N=13)	25 38 (N=6)	Non-drinker		Drinker	
				Exposed children(N=19)	Sibs(N=34)	Exposed children(N=8)	Sibs(N=12)
	No.	No.	No.	No.	No.	No.	No.
Above mean	1	0	2	3	4	0	0
Mean	5	0	2	7	9	0	3
Under mean	0	3	1	1	1	3	3
Mental retardation	0	8	0	3	0	5	0
Subtotal *	6	11	5	14	14	8	6
IQ, Mean	100.3	69.5	103.0	95.4	103.2	82.5	92.5
S.D.	7.2	11.5	12.5	11.8	7.7	5.0	8.2
Comparison	χ${\mathrm{_{{4}}^{{2}}}}$ = 30.8; p < 0.0001			Exposed children			
	Fisher p < 0.0001			χ${\mathrm{_{{3}}^{{2}}}}$ = 9.3, p = 0.02; Fisher p = 0.02			

*Evaluated number of exposed children Bold numbers show significant associations

In addition, we evaluated the cognitive status of the exposed children and their sibs according to the drinking habits of self-poisoned pregnant women (Table 5). Exposed children and their sibs who were born to mothers without a drinking habit had a higher mean IQ than exposed children and their sibs born to alcohol drinking mothers. However, a lower mean IQ was also found in exposed children compared to their unexposed sibs who were born to the same non-drinker mothers and this suggests that there may be a neurotoxic effect of Tardyl®.

Behavioral scales also showed some differences between exposed children and unexposed sibs (Table 4). Of eight mentally retarded exposed children, five had severe and one had moderate behavioral deviation (it was not possible to estimate behavioral status in another mentally retarded boy). In addition two other exposed children had moderate behavioral deviation. On the other hand, of 16 sibs, none had severe and only one had a moderate behavioral deviation.

Thus, of 27 exposed children, 8 were mentally retarded (29.6%) while of 46 sibs, none were mentally retarded (Χ${\mathrm{_{{1}}^{{2}}}}$)=79.7, p<0.0001). Furthermore, moderate behavioral deviations occurred in 2 other exposed children and in one control sib. If we evaluate these 10 exposed children together, the percentage figure is 37.0% in the group of exposed children, while the similar figure was 2.1% in the total group of unexposed sibs based on one child with moderate behavior deviation (OR with 95% CI: 27.6, 3.3-232.4).

Finally we make a comparison of exposed children born to mothers who attempted suicide with Tardyl® and with the components of Tardyl® separately (Table 6). There was no mentally retarded exposed child in the group exposed to amobarbital or glutethimide; one exposed child in the group exposed to promethazine was mentally retarded but the boy had a genetic X -linked fragile X chromosome.Table 7 shows the maternal characteristics in these study groups. The drinking and smoking habits during the study pregnancy were not significantly different in the three components of Tardyl® separately and together in Tardyl®. The mean maternal age and birth order did also not differ significantly among pregnant women who used these drugs and Tardyl® for their suicide attempt.

**Table:** Table 6: **Occurrence of mental retardation, in addition to the distribution of cognitive status with mean IQ and behavioral scale in exposed children born to mothers who attempted suicide during pregnancy with amobarbital, glutethimide, promethazine and Tardyl® (including these three drugs) **

Variables	Amobarbital (N=14)		Glutethimide (N=16)		Promethazine(N=32)		Tardyl®(N=27)	
	No.	%	No.	%	No.	%	No.	%
Mental retardation (instead of MR)	0	0.0	0	0.0	1*	3.1	8	29.6
Cognitive status								
Above mean	0	0.0	1	7.7	5	20.0	3	13.6
Mean	9	81.8	10	76.9	13	52.0	7	31.8
Under mean	2	18.2	2	15.4	6	24.0	4	18.2
MR	0	0.0	0	0.0	1*	4.0	8	36.4
Subtotal	11	100.0	13	100.0	25	100.0	22	100.0
IQ, mean ± S.D.	96.4 ± 8.4		100.4 ± 10.8		98.4 ± 11.2		82.2 ± 20.0		
Behavioral scale								
Normal	5	62.5	8	72.7	11	55.0	4	25.0
Mild	2	25.0	2	18.2	9	45.0	4	25.0
Moderate	0	0.0	1	9.1	0	0.0	5	31.3
Severe	1	12.5	0	0.0	0	0.0	5	31.3
Subtotal	8	100.0	11	100.0	20	100.0	16	100.1

*His brother was also mentally retarded; mental retardation of this sib pair was caused by X-linked fragile X chromosome

**Table:** Table 7: **Comparison of maternal characteristics of pregnant women who attempted suicide with amobarbital, glutethimide and promethazine (i.e. the components of Tardyl®) separately, and Tardyl®**

Variables	Amobarbital(N=14)		Glutethimide(N=16)		Promethazine(N=32)		Tardyl®(N=27)	
Age, yr (mean, S.D.)	22.8	6.1	26.1	7.5	22.9	5.3	22.4	5.4
Birth order (mean, S.D.)	1.5	0.8	2.0	1.3	1.9	1.2	1.6	0.9
Married (%)	50.0		50.0		53.1		63.0	
Socioeconomic status (%)								
High	7.1		12.5		9.4		7.4	
Medium	35.7		37.5		37.5		22.2	
Low	57.1		50.0		53.1		70.4	
Smoker (%)	57.1		43.8		62.5		51.9	
Regular/hard drinker (%)	21.4		31.3		8.8		29.6	

## Discussion

Pregnant women who attempted suicide with Tardyl® showed the general characteristics of self-poisoned pregnant women, i.e. many of them were young, unmarried and had low socioeconomic status, as well as often being smokers and/or drinkers.^10^

Our data did not indicate the “classical” human teratogenic effect of the high doses of Tardyl® (its mean dose was 24-folds higher than the usual clinical dose) because there was no difference in the prevalence at birth of congenital abnormalities between exposed children and their unexposed sibs. The intrauterine fetal development based on pregnancy aged specific birth weight also did not show a significant difference between exposed children and their sibs.

However, of 27 exposed children, eight (29.6%) were diagnosed as mentally retarded and they attended special schools. Mental retardation did not occur among 46 unexposed sibs born to the same mothers. The recorded incidence of mental retardation in school-age (6-18 years) children is about three percent in Hungary,^18^thus the occurrence of mental retardation was about ten-fold higher in exposed children born to the mothers who had attempted suicide with Tardyl®.

Of seven mentally retarded children who were evaluated for behavioral development,5 had severe and one moderate behavioral deviation. In addition, two other exposed children showed moderate behavioral deviation. Thus, of 27 exposed children, ten (37.0%) showed mental retardation and/or severe/moderate behavioral deviation and these findings suggest a possible neurotoxic effect of Tardyl® (i.e., the combination of amobarbital, glutethimide and promethazine).

These results of our study were supported by the lack of familial occurrence of mental retardation in these families. Neither the mothers nor the 46 unexposed sibs of these eight exposed children suffered from any mental retardation. The fathers of exposed children were not examined, but the mothers stated that they did not suffer from mental retardation.

Several environmental agents induce congenital abnormality syndromes (such as FAS, fetal rubella, varicella, hydantoin, valproate, methylmercury effect, iodine deficiency, etc) and these syndromes include both structural defects and mental retardation. In this study we did not find structural defects in the eight exposed children who were mentally retarded. Exposed children and their sibs were examined thoroughly in the study but only FAS was diagnosed: in one exposed boy and one sib, the brother of this exposed boy.

Low birth weight and/or preterm birth is associated with a higher rate of mental retardation and behavioral deviation.^15^Of eight exposed children with mental retardation, two (25.0%) had preterm birth with low birth weight. However, of their 18 sib controls, five (27.8%) had low birth weight. In addition there was no significant difference in the rate of preterm birth and low birth weight between exposed children and sib controls, thus these confounder factors cannot explain the higher risk for mental retardation in exposed children.

In addition, we have to consider an obvious interaction between alcohol abuse and Tardyl®, because five out of the eight exposed children diagnosed with mental retardation were born to drinker mothers. However, the drinking habits may have been exaggerated in this study because pregnant women who attempted suicide with drugs frequently combined this course of action with concomitant alcohol abuse, and these pregnant women were diagnosed as drinkers, though in general they were not hard or regular drinkers. In addition, the phenotypic features of these mentally retarded children did not fit the well-known pattern of FAS.^13,19^FAS was diagnosed only in one exposed boy and in his brother born to the same mother. Exposed children and their sibs were born to the same mothers in general with a similar drinking habit in their pregnancies, nevertheless the mean IQ was significantly lower in exposed children than in their unexposed sib controls born to both the same drinker or non-drinker mothers. On the other hand some interaction between the effect of Tardyl® and alcohol seems to be plausible, but the mental retardation inducing effect of this medicinal product cannot be explained only by the concurrent effect of alcohol.

We did not find a higher rate of mental retardation in exposed children born to mothers who attempted suicide with either amobarbital,^20^ glutethimide^21^or promethazine^22^taken separately, compared with the data of their sibs in the previous studies of these drugs. Thus only the combination of the three drugs in Tardyl® produced a high risk for mental retardation and very low IQ. We hypothesized that the three components of Tardyl® may result in additive or potentiated drug interactions.^23,24^Glutethimide can stimulate hepatic microsomal enzyme production, thus self-induction of its own metabolites, and has frequent interactions with other drugs (e.g., oral anticoagulants) and alcohol.^25^

Another important argument for the potential neurotoxic effect of Tardyl® is that its association with mental retardation in the exposed children occurred when pregnant women attempted suicide with Tardyl® between the 14th and 20th post-conceptional weeks of pregnancy. Otaka and Schull^26^studied the critical period of mental retardation among 1 600 children exposed to radiation from the atomic bomb attack on Japan and found it to be between 8th to 15th post-conceptional weeks. This period corresponds to the time when major proliferation of neuroblasts occurs in the brain of human fetuses.

Thus the final conclusion of this study is that maternal exposure to high doses of Tardyl®, due to the interaction of its component drugs, may induce mental retardation. As far as we know drug induced mental retardation without structural defects has not been described until now.^27,28^

There are many strengths of the self-poisoning model, e.g., there is the potential to estimate a dose-response relationship; one can establish the absence of birth defects after high doses of a drug used during the critical period of embryonic development; there is a potential to examine the neurotoxic effect of drugs following fetal exposure. A further strength of this project is that stresses the clinical and social importance of the self-poisoned pregnant women.^9^

Among the limitations of the study we can mention here that Tardyl® is a popular hypnotic drug in Hungary and some other Central-Eastern European countries, but not in the Western part of Europe. However, the recognition of the potential for a drug effect on the origin of mental retardation appears to be important from both clinical and theoretical points of view.

In conclusion, the results of this study did not show the classical teratogenic, i.e. congenital abnormality inducing effect of high doses of a combination of amobarbital, glutethimide and promethazine (a popular hypnotic drug, Tardyl® in Hungary) in children born to mothers who attempted suicide with this drug during pregnancy. There was no intrauterine growth retardation in children after the suicide attempt of their mothers with high doses of Tardyl®. However, the findings of the study showed a statistically significant association between a higher risk for mental retardation and high doses of Tardyl® when exposure was during the second trimester of pregnancy.
